# Neurotrophic factors as double-edged swords in osteosarcoma: drivers of tumour growth and immune remodelling

**DOI:** 10.3389/fimmu.2025.1666343

**Published:** 2025-10-09

**Authors:** Puzhou Lei, Lei Li

**Affiliations:** Department of Orthopedics Surgery, Shengjing Hospital of China Medical University, Shenyang, China

**Keywords:** neurotrophic factors, osteosarcoma, immune microenvironment, neuro-immune crosstalk, targeted therapy, immunotherapy

## Abstract

Neurotrophic factors, once considered exclusive guardians of neuronal integrity, are increasingly recognised as pivotal regulators of osteosarcoma biology. Their paradoxical enhancement of malignant fitness and an immunosuppressive microenvironment complicates therapy, with metastatic survival remaining stubbornly low. Recent mechanistic studies reveal that ligand-dependent NGF–TrkA, BDNF–TrkB and GDNF–RET circuits intersect with MEK/ERK, PI3K/AKT and STAT3 pathways to ignite proliferation, invasion and metastatic spread. Concurrently, neurotrophin signalling recalibrates macrophage polarity, dampens cytotoxic T-cell function and orchestrates neural-immune feedback loops that shield tumours from surveillance. Harnessing this duality demands an integrative strategy. We synthesise tumour-intrinsic and extrinsic neurotrophic axes, delineate neuro-immune crosstalk, and highlight interventions—TRK/RET inhibitors, CSF1R blockade, β-adrenergic antagonists—aimed at converting this liability into therapeutic leverage. By framing neurotrophic factors as double-edged swords, this review provides a conceptual and practical roadmap for exploiting their vulnerabilities to improve outcomes in osteosarcoma.

## 1 Introduction

Osteosarcoma is the most prevalent primary malignant bone tumour in the paediatric and adolescent population, with a global incidence of roughly 3–5 cases per million each year and a sharp rise during periods of rapid skeletal growth (1–3). Despite multimodal refinements, five-year survival remains ~60–70% (localised) and <30% (metastatic) (4–6). This therapeutic plateau underscores the need for fresh biological insights that can guide innovative treatment strategies.

Classically, the neurotrophin family—NGF, BDNF, NT−3, NT−4/5 and GDNF—governs neuronal survival, axonal guidance and synaptic plasticity (7–9). These proteins signal via high−affinity TrkA/B/C isoforms, the low−affinity p75NTR, and RET–GFRα co−receptor complexes (10, 11). Recent transcriptomic and proteomic surveys of bone tumours have revealed that genes encoding both neurotrophins and their cognate receptors are expressed beyond the nervous system, prompting investigation into their oncological relevance (12, 13).

Accumulating evidence indicates that neurotrophic signalling has tumour-intrinsic consequences in osteosarcoma. HIF-1α drives TrkB transcription in U2OS cells, indicating that the osteogenic niche—defined by hypoxia, high extracellular calcium and constant mechanical remodelling—favours neurotrophin responsiveness via Ca2+-dependent CaMKIV/CREB and calcineurin–NFAT activity, while load-sensing integrin–FAK–YAP/TAZ signalling further primes TrkA/TrkB transcription under metabolic stress (14–16). Furthermore, activation of the BDNF–TrkB axis has been correlated with enhanced proliferation, resistance to apoptosis, and invasive behaviour in multiple malignancies, and similar molecular programmes have been described in experimental osteosarcoma systems (17, 18).

Neurotrophic factors also exert profound effects on non-malignant stromal and immune components. BDNF can skew macrophage polarisation toward an immunoregulatory M2 phenotype and modulate cytokine production, changes that are conducive to tumour immune evasion (19, 20). More broadly, neurotrophin-mediated cross-talk between peripheral nerves and immune cells has been implicated in shaping the inflammatory milieu of several solid tumours, a concept that is increasingly explored in bone sarcomas (21–23).

These observations highlight the context-dependent, bifunctional behaviour of neurotrophic factors in osteosarcoma: they can directly enhance malignant cell fitness while simultaneously reprogramming the host immune landscape. Deciphering this duality is therefore critical for the rational deployment of targeted and immunomodulatory therapies. We define tumour−intrinsic edges as neurotrophin actions within cancer cells and tumour−extrinsic edges as effects on immune or stromal cells; this review accordingly addresses (1) intrinsic drivers, (2) extrinsic modulation, and (3) neuro−immune feedback. By integrating these dimensions, we aim to identify therapeutic vulnerabilities that may convert this biological liability into a clinical opportunity. We searched PubMed and ClinicalTrials.gov (2010-June 2025) using ‘osteosarcoma’, ‘neurotrophin/NGF/BDNF/RET/Trk’, and ‘immunity’; English; prioritised primary OS data then mechanistic studies in other tumours; excluded reviews/abstract-only reports; key outcomes included pathway activity, immune metrics, and clinical responses.

## 2 Tumour-intrinsic edge: neurotrophic drivers of osteosarcoma growth, survival and metastasis

Large-scale interrogation of TARGET-OS and tissue micro-arrays shows osteosarcoma cells actively express neurotrophic ligands and cognate receptors (24, 25). NGF–TrkA shows highest expression; BDNF–TrkB and GDNF–GFRα/RET are variable, whereas NT−3/TrkC is low in osteosarcoma but prominent in other bone sarcomas, hence our emphasis (26–28). Expression patterns correlate with clinicopathological variables such as stage and metastatic propensity, indicating biological rather than incidental relevance.

The NGF–TrkA axis exemplifies an autocrine–paracrine circuit that reinforces malignant fitness. NGF stimulation in 143B and MG-63 cells triggers rapid MEK/ERK phosphorylation followed by transcriptional up-regulation of MMP-2, thereby accelerating wound closure, Transwell invasion and experimental lung colonisation (29, 30). Pharmacologic Trk blockade or MEK/ERK silencing abrogates these effects *in vitro* and reduces metastatic burden in orthotopic xenografts, underscoring pathway druggability (31, 32). Mechanistically, NGF lowers miR-92a-1-5p, lifting repression of MMP-2 and creating a feed-forward loop that promotes extracellular-matrix remodelling (33, 34). Such findings extend earlier observations that hypoxia inducible factors maintain TrkA transcription under metabolic stress and suggest that NGF signalling provides a selectable advantage in the poorly vascularised bone niche.

Although expressed at lower levels, BDNF-TrkB signalling confers distinct survival benefits. Epithelial models show TrkB-driven anoikis resistance via PI3K/AKT; osteosarcoma evidence remains preclinical, with similar signatures in cell lines and xenografts (35, 36). Osteosarcoma cells appear to reuse this programme: exogenous BDNF enhances clonogenicity, mitigates chemotherapy-induced apoptosis and promotes a spindle-shaped, vimentin-positive phenotype, features that collectively align with heightened metastatic risk (37, 38). Down-stream, TrkB engages PI3K/AKT, PLCγ/PKC and, in nutrient-limited settings, STAT3, integrating prosurvival and metabolic rewiring (39, 40). The plasticity of this circuitry rationalises investigations combining conventional cytotoxics with ATP-competitive TRK inhibitors or AKT antagonists.

The GDNF–GFRα1–RET module adds another layer of complexity. Osteosarcoma sub-clones with elevated GFRα1 expression demonstrate increased motility, drug tolerance and mesenchymal marker expression; conversely, GFRα1 interference restores chemosensitivity (41, 42). Soluble GFRα1, released by adjacent Schwann cells, can activate RET in trans, linking perineural niche signalling to malignant dissemination, a phenomenon already recognised in pancreas and prostate cancer (43, 44). RET activation funnels into the MAPK and JAK/STAT cascades, converging on transcriptional regulators that control cell-cycle progression and oxidative-stress resilience, characteristics advantageous for survival in circulation and at secondary sites.

Genomic alterations further amplify neurotrophic signalling. Oncogenic NTRK1/2/3 fusions—often retaining an active kinase domain—are documented but rare in osteosarcoma cohorts. These lesions confer exquisite sensitivity to first-line TRK inhibitors such as larotrectinib and repotrectinib, mirroring tissue-agnostic responses observed across solid malignancies (45, 46). The occurrence of such fusions, together with ligand-dependent activation described above, positions neurotrophin-RTK signalling as a central vulnerability that can be exploited both genomically and pharmacodynamically.

Taken together, NGF-TrkA, BDNF-TrkB and GDNF–RET circuits endow osteosarcoma cells with proliferative, anti-apoptotic and pro-metastatic properties. Convergence on MEK/ERK, PI3K/AKT/mTOR and JAK/STAT modules offers multiple pharmacological checkpoints, while the demonstrable efficacy of TRK inhibitors in fusion-positive disease highlights the clinical translational potential.

## 3 Tumour-extrinsic edge: neurotrophic remodelling of the osteosarcoma immune microenvironment

The cellular and acellular constituents of the osteosarcoma milieu are highly sensitive to neurotrophic cues that originate from malignant cells, infiltrating nerves and, to a lesser extent, resident stromal elements (47, 48). Single-cell deconvolution of primary tumours, coupled with spatial omics, reveals TrkA/TrkB/RET programmes in TAMs, dendritic and endothelial cells and maps their adjacency to nerve fibres and malignant cells, indicating that the immune landscape is intrinsically wired to perceive nerve-derived growth factors (49, 50). Functionally, NGF released by osteosarcoma cells up-regulates ICAM-1/VCAM-1 on circulating monocytes, accelerates extravasation, and skews differentiation toward an M2 phenotype via TrkA–ERK signalling (51, 52). BDNF–TrkB engagement on CD8^+^ T cells (shown in other tumours) dampens effector cytokines and increases PD-1 via PI3K/AKT-dependent re-programming; OS data are limited (53, 54). GDNF signalling through the GFRα1/RET complex in myeloid cells activates STAT3, enhances IL-10 secretion and impairs antigen presentation, a constellation that favours immune escape (55, 56). These observations mirror broader oncological evidence that neurotrophic factors orchestrate a permissive, low-immunogenic niche by synchronising axonogenesis with immune suppression. The principal mechanisms operative in osteosarcoma are summarised in **Table 1**.

**Table 1:** Neurotrophic-factor–driven immunologic remodelling in the osteosarcoma microenvironment.

Neurotrophic factor	Primary immune/stromal target(s)	Cognate receptor(s) on target	Key downstream signalling outcome	Immunological consequence
NGF	Circulating monocytes → TAMs	TrkA/p75^NTR^	ERK-mediated VCAM-1/ICAM-1 induction; STAT6 activation	Enhanced M2 polarisation, IL-10 and TGF-β release, phagocytosis dampening
BDNF	Effector T cells (CD8^+^, CD4^+^)	TrkB	PI3K/AKT-driven PD-1 up-regulation; reduced granzyme transcription	Cytotoxic attenuation, promotion of T-cell exhaustion
NT-3	Endothelial progenitors	TrkC	MAPK activation, VEGF-A co-secretion	Endothelial activation, pro-angiogenic loop that indirectly limits immune infiltration
GDNF	Myeloid‐derived suppressor cells/TAMs	GFRα1–RET complex	STAT3 phosphorylation; ROS detoxification	Expansion of suppressive myeloid subsets and antigen-presentation deficit
Pro-neuronal exosomes (miR-21/miR-34a-rich)	Schwann-like stromal cells	Endocytic uptake (non-canonical)	Schwann-cell activation, axon guidance molecule release	Physical nerve ingrowth that reinforces neuro-immune signalling

Neurotrophin-responsive immune cells constitute a feed-forward circuit in which axonal infiltration, cytokine skewing and checkpoint induction converge to insulate osteosarcoma from effective immunosurveillance. Because PD-1/PD-L1 monotherapy yields <10% responses in relapsed osteosarcoma, interrupting this circuit via TRK/RET inhibition or macrophage re-education may unlock checkpoint synergy.

## 4 Bidirectional crosstalk: feedback loops linking neurotrophic signalling and immunity

Emerging evidence indicates that neurotrophins and immune mediators in osteosarcoma engage in tightly inter-connected positive feedback loops that reinforce both malignant cell fitness and micro-environmental immune suppression (57, 58). Osteosarcoma cells secrete NGF, BDNF and related cues that attract peripheral nerves and condition infiltrating leukocytes; reciprocally, cytokines and chemokines released by those stromal elements amplify neurotrophic signalling, thereby establishing a self-perpetuating circuit rather than a unidirectional pathway (59, 60). Dense axonal ingrowth driven by tumour-derived NGF has been associated with increased PD-1 expression on local T lymphocytes and a rise in M2-polarised macrophages, illustrating how neuronal inputs synchronise with immune checkpoints to maintain an immunosuppressive milieu.

**Figure 1:**
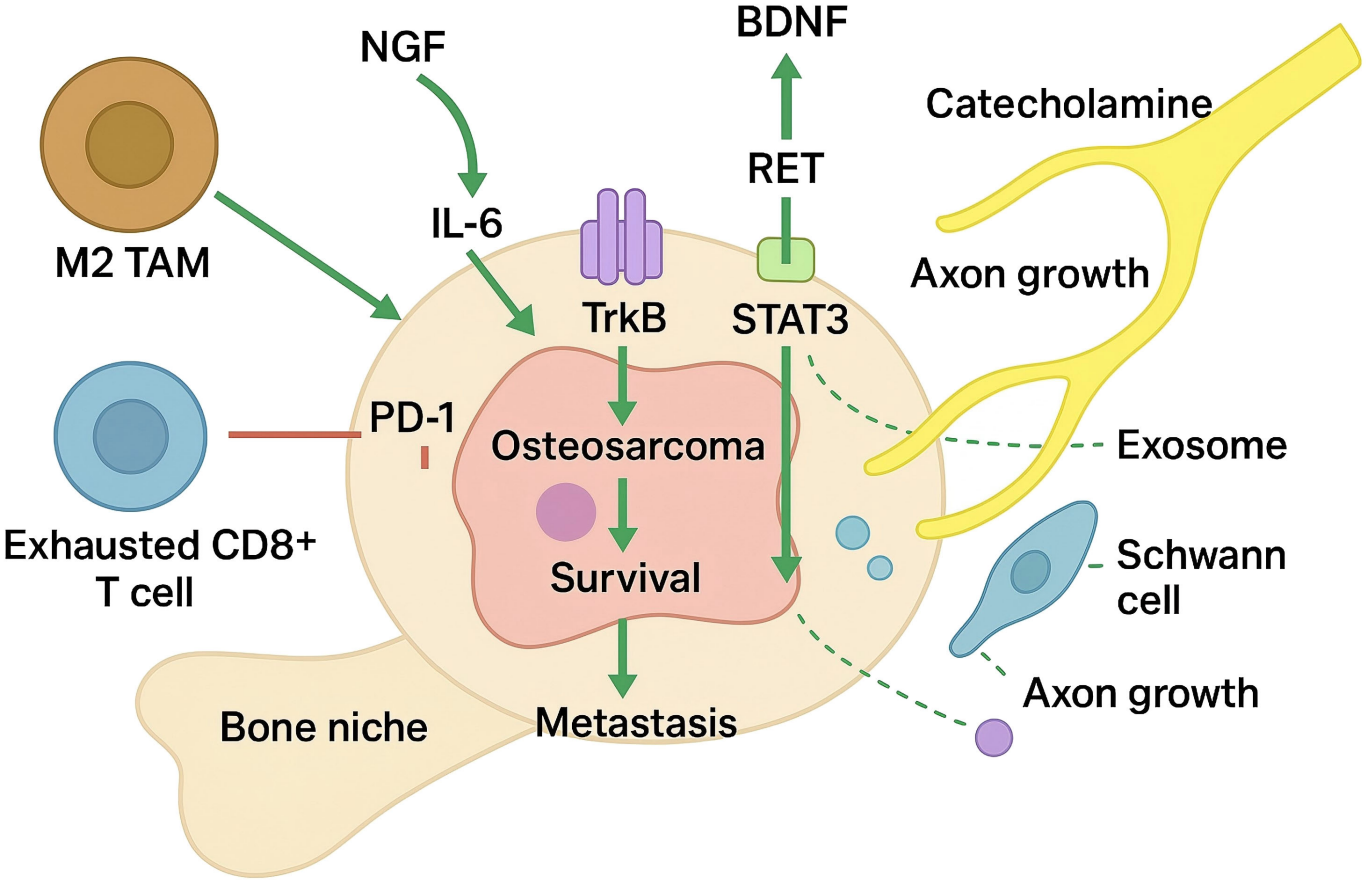
Neurotrophic factor-driven neuro-immune feed-forward loops in the osteosarcoma bone niche.

As shown in **Figure 1**, cytokine induction of neurotrophins represents a first tier of reciprocity. Interleukin−6 from Schwann−like stromal cells or M2 macrophages activates STAT3, which partners with phosphorylated TrkB at BDNF enhancers, forming a feed−forward loop that amplifies neurotrophin output and tumour aggressiveness (61, 62). Similar IL-1β and TNF-α inputs have been reported to modulate NGF and BDNF levels in bone-associated tumours, suggesting a broader principle whereby inflammatory mediators act as upstream rheostats of neurotrophic output.

A second tier comprises neurotrophin-driven chemokine gradients that reinforce immune cell influx. BDNF/TrkB activation in solid tumours triggers JNK-dependent CCL2, recruiting CCR2^+^ monocytes that become TAMs and secrete IL-10, TGF-β and IL-6, sustaining TrkB phosphorylation; OS validation is pending (63, 64). Psychological stress elevates norepinephrine/epinephrine that engage β2-adrenergic receptors (ADRB2) on tumour and immune cells, raising cAMP to activate PKA and Epac→Rap1/JNK: PKA-phospho-CREB transactivates BDNF promoter IV, while JNK/AP-1 and STAT3 signalling increase CD274 (PD-L1) transcription; ADRB2 blockade (e.g., propranolol) reverses these changes and attenuates CCL2-mediated myeloid influx.

Macrophage‐derived NGF adds an additional layer of amplification. In the osteogenic bone niche, tumour-conditioned macrophages and osteoclast precursors can synthesise NGF, which in turn enhances ICAM-1-mediated monocyte adhesion and supports further macrophage infiltration (65, 66). The ensuing rise in local NGF concentration potentiates TrkA signalling in both malignant cells and nerve terminals, escalating axonal density and reinforcing macrophage tropism in a feed-forward manner.

Extracellular vesicles act as mobile amplifiers within these circuits. Osteosarcoma-derived exosomes enriched in miR-21 and miR-34a—often accompanied by miR-181a/miR-222/miR-146a—activate Schwann cells (via ERK–c-Jun) to secrete guidance cues and proNGF/GFRα1, stimulating neurite extension; in immune cells, exosomal miR-21 targets PTEN/PDCD4 and miR-146a modulates TRAF6–NF-κB to skew TAMs toward suppressive states, while Schwann-cell CCL2/CXCL5 recruits CCR2^+^ myeloid populations that stabilise the neuro-immune niche (67, 68). These multilayered feedback loops integrate neuronal, immune and malignant compartments into a cohesive signalling circuit that simultaneously accelerates osteosarcoma progression and dampens effective antitumour immunity. Therapeutic disruption of any single node—such as β-adrenergic blockade, Trk or RET inhibition, STAT3 antagonism, or CCL2/CCR2 axis interference—may therefore propagate inhibitory effects throughout the entire network, offering a rationale for combinatorial strategies that target both neurotrophic and immunological facets of the disease.

## 5 Harnessing neurotrophic duality: integrative therapies targeting tumour growth and immune remodelling

Pharmacological blockade of neurotrophin receptors now constitutes the most direct strategy to exploit the tumour-intrinsic arm of the NGF–TrkA/BDNF–TrkB axis while simultaneously attenuating the immunoregulatory feedback loops described above (69, 70). Larotrectinib, an ATP-competitive inhibitor with nanomolar affinity for all Trk isoforms, curtailed orthotopic osteosarcoma expansion and abolished experimental lung metastases in a murine model driven by NGF over-expression, an effect accompanied by reduced MEK/ERK activity and restoration of miR-92a-1-5p levels (71, 72). Although approvals are fusion-agnostic, ligand-dependent OS may also benefit; this remains hypothesis-generating and should be tested with pharmacodynamic readouts (e.g., p-TRK/RET) while accounting for niche modifiers (hypoxia, Ca2^+^, YAP/TAZ) (73, 74). RET-selective inhibitors such as selpercatinib have demonstrated durable responses in solid tumours bearing RET rearrangements; pre-screening of relapsed osteosarcoma for rarer NTRK or RET fusions therefore represents a rational enrichment strategy for precision trials that marry cytostatic and immunomodulatory endpoints.

Targeting neurotrophin-conditioned myeloid compartments is equally critical. *In vivo* administration of the CSF1R inhibitor pexidartinib (PLX3397) depleted M2-polarised macrophages, increased intratumoural CD8^+^ T-cell density and suppressed both primary tumour growth and pulmonary dissemination in orthotopic and patient-derived xenografts of osteosarcoma (75, 76). CSF1R blockade de-phosphorylates ERK and flips ARG1^+^ M2 macrophages to iNOS^+^ M1 via STAT6 loss, restoring MHC-II, CD86 and IL-12, enhancing cross-presentation to CD8^+^ T cells and thereby potentiating PD-1/PD-L1 antibodies in addition to reinforcing TRK inhibition through MAPK suppression and increased phagocytosis (77, 78). Given that NGF and BDNF transcription correlate positively with CSF1 expression in bulk RNA-seq datasets, sequential or combined Trk/CSF1R inhibition could interrupt two nodes of the same positive-feedback circuit and warrants formal evaluation.

Stress-responsive β-adrenergic signalling represents a third actionable layer linking neural inputs to immune suppression. Non-selective β-blockade with propranolol reduced osteosarcoma proliferation, impaired angiogenesis and potentiated low-dose cisplatin in xenograft models. Beyond cytostasis, propranolol enhanced T-cell infiltration and lowered myeloid-derived suppressor cell burden in syngeneic soft-tissue sarcoma, thereby amplifying the efficacy of anti-CTLA-4 therapy without additive toxicity (79, 80). Because catecholamine release is heightened by tumour-induced neo-innervation, β-adrenergic blockade may act upstream of the neurotrophin–immune loop, providing a low-cost adjunct to both kinase inhibition and checkpoint blockade.

These observations argue for multi-axis regimens (hypothesis-generating) that attenuate neurotrophin receptors, re-educate macrophages and dampen sympathetic inputs. Proposed sequence (for testing): molecular screening; short-course TRK/RET inhibition to debulk; add CSF1R blockade to shift macrophages; consider β-blockade plus PD-1/PD-L1 for maintenance; monitor neuropathy, hepatic AEs, QT, and paediatric growth-plate changes. Pharmacodynamic read-outs—p-TRK/RET, M2/M1 ratios, intratumoural catecholamines, IFN-γ signatures—should guide early-phase trial ordering and dosing. OS cohorts report low PD-1/PD-L1 monotherapy responses, rare NTRK/RET fusions, and ongoing OS-relevant trials testing TRK/RET inhibitors, CSF1R agents, and β-blockers with immunotherapy. The availability of paediatric-friendly formulations of larotrectinib and selpercatinib, the manageable safety profile of propranolol, and emerging oral CSF1R inhibitors create a realistic path toward combination protocols that convert neurotrophic duality from a liability into a therapeutic lever.

## 6 Translational outlook: targeting the double-edged sword for therapeutic gain

Understanding that neurotrophic factors can simultaneously accelerate malignant growth and subvert antitumour immunity places their signalling nodes among the most attractive, yet challenging, translational targets in osteosarcoma. The first prerequisite for clinical progress is molecular stratification that distinguishes tumours driven by ligand-dependent NGF-TrkA, BDNF-TrkB or GDNF–RET loops from those harbouring activating rearrangements or kinase-domain point mutations. Immunohistochemistry, RNA-seq–derived expression scores and DNA-based fusion panels are already feasible in routine pathology and should be embedded prospectively in early-phase studies to enrich for pharmacologically tractable subsets and to provide correlative datasets linking pathway dependence with immunological contexture (60, 65).

Drug-development pipelines are advancing beyond first-generation ATP-competitive inhibitors. Next−gen agents such as selitrectinib and zurletrectinib remain active against xDFG and solvent−front (G595R, G667C) TRK mutations while retaining CNS penetration (72, 77). Parallel efforts are refining highly selective RET inhibitors with favourable paediatric safety profiles and negligible off-target VEGFR blockade, a characteristic that may attenuate dose-limiting hypertension and thrombotic events often observed with multikinase agents (41). Adaptive designs with real-time PD (p-TRK/RET suppression, circulating neurotrophins, TAM polarisation) are recommended.

Translational leverage will increase further when kinase inhibition is combined with rational immunomodulation. CSF1R blockade remodels macrophage composition, reduces IL-10/TGF-β output and indirectly attenuates Trk-driven ERK activity in malignant cells, providing a mechanistic basis for dual CSF1R–TRK schedules (46). β-adrenergic antagonists diminish catecholamine-induced BDNF up-regulation and enhance CD8^+^ T-cell infiltration, making them logical, low-toxicity partners for neurotrophin-axis inhibitors or for anti-PD-1/PD-L1 antibodies once target engagement has curtailed tumour-intrinsic growth signals (18, 29). Because STAT3 integrates both neurotrophic and cytokine cues, early introduction of selective STAT3 degraders might suppress parallel survival pathways and blunt feedback re-activation observed with single-agent kinase therapy (70). Selecting the optimal sequence—kinase inhibition to debulk disease, macrophage re-education to disarm immune suppression, checkpoint blockade to sustain cytotoxic surveillance—will likely require window-of-opportunity trials with paired biopsies and multiplex spatial profiling.

Heterogeneity of neuro-immune coupling mandates preclinical test systems that faithfully recapitulate bone, neural and immune compartments. Three-dimensional tri-culture platforms and biomimetic scaffolds permit interrogation of neurite outgrowth, macrophage plasticity and T-cell trafficking under defined gradients of NGF, BDNF and catecholamines (58). Integration of single-cell and spatial transcriptomics from primary tumours and matched patient-derived xenografts exposes micro-niches in which neurotrophic signalling is dominant and predicts regional variability in drug response (9, 48). Such datasets will be invaluable for constructing computational models that nominate combination regimens and identify early biomarkers of benefit or resistance.

Toxicity management must evolve in parallel with therapeutic complexity. Careful neuro−cognitive and growth−plate monitoring is essential because paediatric TRK or RET inhibitor trials report mostly grade−1/2 neuropathy, transient liver enzyme rise, weight gain, physeal thickening and occasional hypertension—usually reversible with dose adjustment (5, 26). Off-tumour macrophage depletion, catecholamine withdrawal and cytokine shifts will necessitate longitudinal immune monitoring to pre-empt opportunistic infections or auto-inflammatory sequelae.

These considerations delineate a roadmap in which precise molecular selection, next-generation kinase inhibitors, and context-specific immunomodulators converge to neutralise both arms of the neurotrophic double-edged sword. Successful implementation will hinge on integrative trial designs that capture pharmacodynamic, immunological and functional imaging endpoints, enabling rapid iteration toward durable, low-toxicity control of osteosarcoma.
